# Validation of a Pseudovirus Neutralization Assay for Severe Acute Respiratory Syndrome Coronavirus 2 Omicron JN.1 and LP.8.1 Subvariant Lineage Strains with Homologous and Heterologous Matched Sera in Clinically Relevant Samples

**DOI:** 10.3390/microorganisms14051042

**Published:** 2026-05-05

**Authors:** Zhaohui Cai, Raj Kalkeri, Benjamin Haner, Mi Wang, Paul Skonieczny, Bahar Osman, Dominic Dent, David Silva, Kevin Auerbach, Emmanuel Faust, Sheau-Line Feng, Miranda R. Cai, Mingzhu Zhu, Shane Cloney-Clark, Joyce S. Plested

**Affiliations:** Clinical Immunology, Novavax, Gaithersburg, MD 20878, USA

**Keywords:** COVID-19, immunogenicity, SARS-CoV-2, assay validation, correlate of protection, Omicron JN.1

## Abstract

The pseudovirus neutralization (PNT) assay is an established high-throughput, robust, and efficient BSL-2 method for detecting neutralizing antibodies (NAbs) against SARS-CoV-2 with the correlates of protection previously established for the ancestral (Wuhan) strain. The PNT assay was validated using nonmatched ancestral sera with anti-JN.1 cross-NAbs, clinically matched JN.1 sera with anti-JN.1 NAbs, or nonmatched JN.1 sera with anti-LP.8.1, KP.2, or KP.3 cross-reacting NAbs. In line with predefined validation acceptance criteria, the PNT assay was precise, with %GCV ≤ 50 in ~90–100%/200 results (40 samples/strain). The acceptance criteria were met for linearity (slope ranged from 1.041 for ancestral sera with anti-JN.1 NAbs to 1.213 for JN.1 sera with anti-KP.2 NAbs), *R*^2^ (0.9619–0.9944 for ancestral sera with anti–JN.1 NAbs), % relative bias, and total %GCV < 50 for almost all of the 15 serum samples tested for four virus strains. Human sera collected pre–COVID-19 had no detectable titer for tested Omicron JN.1 subvariants (<LLOQ) and all influenza and RSV clinical samples tested negative (<LLOQ) for SARS-CoV-2 and highly immunogenic for seasonal influenza or RSV post-vaccination, demonstrating the PNT assay specificity. Our data suggest this assay is suitable for assessing immune responses to ancestral and current SARS-CoV-2 strains and has potential for evaluating cross-reacting NAbs against emerging Omicron JN.1 subvariants.

## 1. Introduction

Neutralizing antibodies (NAbs) developed following a SARS-CoV-2 infection or vaccination, or a combination of both, act as correlation markers to assess vaccine efficacy [1,2]. Measuring the levels of neutralizing antibodies specific to SARS-CoV-2 in part facilitates tracking the effectiveness of anti-viral vaccines [3] while recognizing that the record of NAb levels reflects not only natural infection but also the NAb response to prior vaccination. The pseudovirus neutralization (PNT) assay is a high-throughput, robust, and efficient method for detecting neutralizing antibodies against the SARS-CoV-2 spike protein [4] and was reported previously to measure neutralizing antibodies against SARS-CoV-2 Omicron BA.1, BA.2, and BA.5 subvariants [5]. Another study [5] validated the PNT assay for measuring neutralizing antibody titers against Omicron subvariants BA.5 and XBB.1.5. The results demonstrated the assay’s utility in analyzing clinical trial samples to assess the immunogenicity of SARS-CoV-2 vaccines against a series of COVID-19 variants.

Given the recommendations to include JN.1 lineages in the updated 2024–2025 COVID-19 vaccines [6,7], there is an urgent need for a validated PNT assay to rapidly determine vaccine immunogenicity using specific matched homologous and/or cross-reactive heterologous serum samples from clinical trials against the circulating Omicron JN.1 subvariant and its descendants. Here, we describe the validation of the PNT assay for detecting and quantifying the neutralizing antibodies against SARS-CoV-2 Omicron JN.1 lineage (JN.1, LP.8.1, KP.2, KP.3) subvariants, to demonstrate its suitability for testing serum samples from COVID-19 vaccine trials. The assay validation was based on pre-defined parameters for precision, linearity, and specificity. Assay lower (LLOQ) and upper limits of quantitation (ULOQ) were also determined. Additionally, to demonstrate cross-reactivity, the neutralizing activity of serum samples from participants in the 2019nCoV-311 part 2 (ClinicalTrials.gov NCT05372588) vaccine trial was assessed against the SARS-CoV-2 Omicron JN.1 lineage (LP.8.1, XEC, KP.2, KP.3, KP.3.1.1) subvariants, using the PNT assay.

## 2. Materials and Methods

### 2.1. Assay Procedure

The PNT validation assay method described previously [4,5] was further developed to evaluate the presence of virus-specific and cross-reactive neutralizing antibodies against the variant strains of SARS-CoV-2 in human serum. Briefly, human serum samples were first heat-inactivated for 30 min at 56 °C to destroy the serum complement. Each heat-inactivated serum sample was then diluted 1:10 in an infection medium. An equal volume of working dilution of pseudovirus (eENZYME, LLC, Gaithersburg, MD, USA) was then added to each well, followed by incubation at 37 °C for 2 h. Then, 1.0 × 10^4^ HEK293T/hACE2 cells (Creative Biogene, Shirley, NY, USA) in 100 µL of infection medium were added to the wells, followed by incubation for 72 h at 37 °C. After incubation, 50 µL BrightGlo Luciferase substrate (Promega, Fitchburg, WI, USA) was added to each well. Plates were incubated for 15 min at room temperature without ambient light. Viral entry into the cells was determined by measuring the luminescence with a SpectraMax iD3 microplate reader (Molecular Devices, San Jose, CA, USA). The inhibitory dilution of serum samples at which relative luminescence units were 50% lower than virus control wells on each plate represented PNT titers. Data were analyzed and 50% PNT titers (ID50) were calculated using a four-parameter curve fitting in SoftMax^®^ Pro software 7.1.1 (Molecular Devices, San Jose, CA, USA).

### 2.2. Samples and Controls

Human serum samples used were mainly in-house prepared samples, generated by pooling two or more individual serum to obtain sufficient volume from the Novavax COVID-19 vaccine trials 2019nCoV-315 (ClinicalTrials.gov NCT06409663), 2019nCoV-301, (ClinicalTrials.gov NCT04611802), or 2019nCoV-311 part 2 (ClinicalTrials.gov NCT05372588) [8,9], the Novavax quadrivalent seasonal influenza vaccine trial (qNIV E 301) [10] (Shinde V, et al., 2022), and from the Novavax respiratory syncytial virus (RSV) vaccine maternal trial (Novavax; RSV-M-301) [11]. Some human serum samples used were commercial samples that had shown positive and/or negative SARS-CoV-2 PNT titers during the screening process for NAbs (BioIVT, Woodbury, NY, USA, or Biological Specialty Co., Colmar, PA, USA).

Positive and negative serum samples were included in each assay to serve as quality controls. Positive control samples were human serum samples with a high, medium, or low range of PNT titers. The SARS-CoV-2 antibody-negative serum (BioIVT, Woodbury, NY, USA) sample served as a negative control. The details of serum samples tested in the PNT validation assay for Omicron JN.1, KP.2, and KP.3 subvariants are listed in Supplementary Table S5.

### 2.3. Validation Assays

#### 2.3.1. Precision

Intra- and inter-assay precision were assessed using a panel of 40 human serum samples that ranged from negative as well as low-to-high PNT titers. The assays were performed in six runs by different analysts on different days, with each sample tested in duplicate and twice per run following standard harmonized validation practices [12]. The intra- and inter-assay precision were assessed by calculating the percent of the geometric coefficient of variation (%GCV) using the variance component analysis model with the sample as the fixed effect and the analyst and day as random effects. The %GCV was estimated based on the natural log-transformed values of PNT titers. Precision data were evaluated at both the individual sample and strain level, which formed the overall assay variance of all 40 samples tested for each variant. The acceptance criterion of %GCV for geometric mean titer (GMT) results was ≤50% for ≥80% of positive samples.

#### 2.3.2. Linearity

The linearity of the Omicron JN.1 subvariant PNT assay was determined by testing more than two SARS-CoV-2 PNT-positive human serum samples undiluted or diluted independently at different dilution factors. The samples were tested in duplicate, twice in a run across six runs, by two different analysts on three days for each subvariant. Similarly, to confirm the assay linearity for Omicron LP.8.1, KP.2, and KP.3 subvariants, an additional two samples for each subvariant were tested in duplicate, twice in a run across six runs, by different analysts on different days. For each dilution, the samples were diluted independently in a negative serum, followed by heat inactivation. The expected titer at each dilution was calculated from the overall GMT of the undiluted sample from all runs divided by the dilution factor of each dilution for each sample. The observed PNT titer at each dilution was the overall GMT at each dilution for each sample in all runs.

A linear regression analysis was then performed on the sample results. The independent variable was log10 expected PNT titer and the dependent variable was log10 observed PNT GMT. The slope point estimate and 95% confidence interval (CI) of the regression line slope were evaluated, and the coefficient of determination (*R*^2^) was estimated. The percent (%) relative bias was also estimated at each dilution for each sample. The acceptance criterion was >80% of the dilution points for each sample having a % relative bias between −60% and 150% for relative accuracy and %GCV ≤ 50 for precision. The acceptance criterion for slope was between 0.70 and 1.43 and the *R*^2^ was ≥0.95.

#### 2.3.3. Specificity

The specificity of the PNT assay was assessed using samples collected in the pre–SARS-CoV-2 pandemic era and samples notable for strong specific immune responses against other pathogens such as Seasonal Influenza and RSV. Five pairs of pre- and post-vaccination serum samples from the seasonal influenza vaccine trial [10] and five pairs of pre- and post-vaccination serum samples from the RSV vaccine trial [11] were assessed in the PNT assay. Additionally, eight normal healthy human serum samples collected in the pre–SARS-CoV-2 pandemic era were tested in the PNT assay for each variant.

#### 2.3.4. Assay of Analytical Range

The lower limit of quantitation (LLOQ) and upper limit of quantitation (ULOQ) of the PNT assay were determined based on the precision data and the % relative bias of the linearity tests. The overall GMT, %GCV, and % relative bias of each dilution of each sample were calculated. The lowest and the highest PNT titer ranges that achieved acceptable precision, accuracy (% relative bias), and maintained linearity for the linearity test samples (JN.1^a^, *n* = 3, JN.1^b^ *n* = 2, KP.2/KP.3, *n* = 4, and LP.8.1, *n* = 2) were designated as the LLOQ and ULOQ, respectively.

Note: JN.1^a^ refers to ancestral Wuhan non-matched (‘heterologous’) sera with cross-neutralizing antibodies against the Omicron JN.1 subvariant, while JN.1^b^ refers to JN.1-matched (‘homologous’) sera with neutralizing antibodies against the Omicron JN.1 subvariant.

### 2.4. Cross-Reactivity of 2019nCoV-311 Part 2 Serum Samples Against Omicron JN.1 Lineage (KP.2, KP.3, KP.3.1.1, XEC, and LP.8.1) Subvariants

We previously analyzed the cross-reactivity of neutralizing antibody responses of a monovalent Omicron JN.1 subvariant vaccine against multiple SARS-CoV-2 pseudovirus strains using both validated (ancestral Wuhan strain and Omicron BA.5, XBB.1.5, JN.1, KP.2, and KP.3 subvariants) and fit-for-purpose (Omicron KP.3.1.1, XEC, MC.1, LP.8.1, LF.7.2.1, LF.7.7.2, NB.1.8.1, XFC, and XFG subvariants) assays [13] (Alves K, et al., 2026). To further evaluate the clinical utility of the PNT assay, serum samples (*n* = 12) from participants in the 2019nCoV-311 part 2 vaccine trial (Wuhan, Omicron BA.5 subvariant, and bivalent Wuhan and Omicron BA.5 subvariant vaccines) were tested to assess cross-reactivity against 6 Omicron subvariants (JN.1, KP.2, KP.3, KP.3.1.1, XEC, and LP.8.1) (Supplementary Figure S1). The neutralizing antibody responses (ID50) were characterized as GMTs and their 95% CIs.

### 2.5. Statistical Analysis

Statistical analyses were performed using statistical analysis software (SAS^®^) version 9.4 (SAS Institute Inc., Cary, NC, USA) in a Windows environment. The cross-reactivity potential of the serum samples was determined using the one-way analysis of variance test followed by Dunn’s multiple comparisons test.

## 3. Results

The PNT validation analysis described here was expected to meet validation criteria when performed using either nonmatched (‘heterologous’) ancestral Wuhan sera with cross-neutralizing antibodies against the Omicron JN.1 subvariant, specific clinically matched (‘homologous’) JN.1 sera with neutralizing antibodies against the Omicron JN.1 subvariant, or nonmatched (‘heterologous’) JN.1 sera with cross-reacting neutralizing antibodies against the Omicron LP.8.1, KP.2, and KP.3 subvariants [13].

### 3.1. Assay Validation Parameters

#### 3.1.1. Precision

Overall intra-assay, inter-assay, and total assay variability values for %GCV obtained for non-matched (‘heterologous’) ancestral Wuhan sera with cross-neutralizing antibodies against the Omicron JN.1 subvariant were 28.9, 21.5, and 36.6, respectively (Table 1). At the individual sample level, among all 40 samples, the lowest %GCV value for each assay was 0.0, and the highest %GCV values were 37.7, 44.9, and 61.0, respectively. As expected, the percentage of individuals with assay precision results that met the targeted acceptance criteria of %GCV ≤ 50 was high (i.e., 100, 100, and 87.5, respectively) (Table 1).

These values for the precision parameters were generally similar to those for specific clinically matched (‘homologous’) JN.1 sera with neutralizing antibodies against the Omicron JN.1 subvariant (Table 1) and to those for non-matched (‘heterologous’) JN.1 sera with cross neutralizing antibodies against the Omicron LP.8.1 subvariant (Table 1) and Omicron KP.2, and KP.3 subvariants (Supplementary Table S1).

#### 3.1.2. Linearity

**Figure 1:**
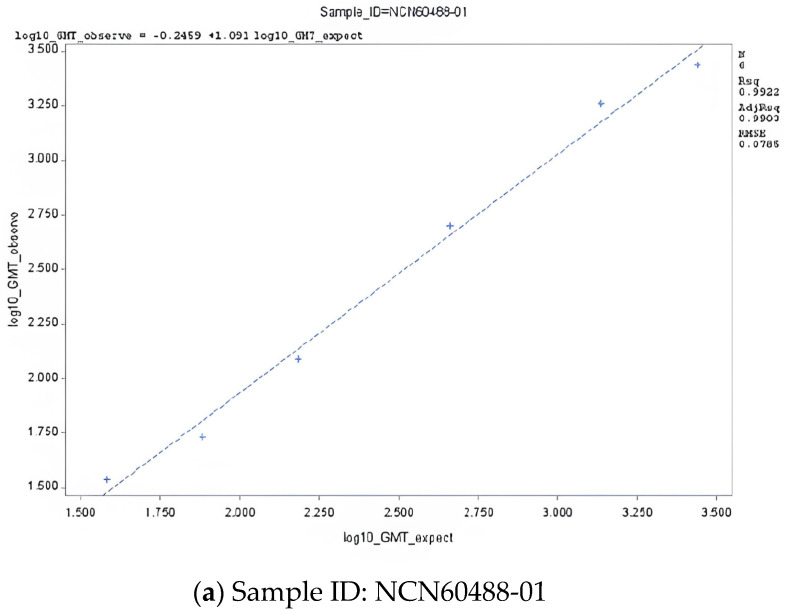

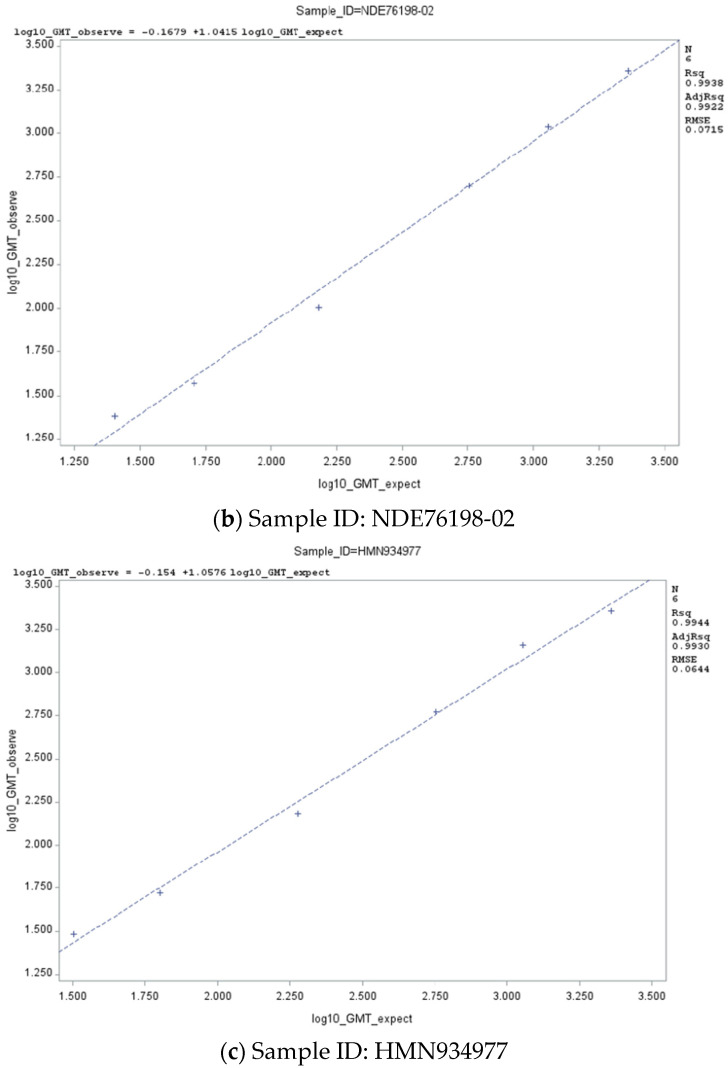
Linear regression plot of PNT GMT against Omicron JN.1 subvariant—non-matched (‘heterologous’) ancestral Wuhan clinical sera. Abbreviations: GMT, geometric mean titer; ID, identification; PNT, pseudovirus neutralization.

A linearity analysis of the PNT assay was performed using three non-matched (‘heterologous’) ancestral Wuhan sera with cross-reacting neutralizing antibodies against the Omicron JN.1 subvariant. All three serum samples met the targeted acceptance criteria for slope and *R*^2^ (Table 2). The linear regression plots are presented in Figure 1a–c. All dilution levels (100%) in all three serum samples had % relative bias between −60 and 150, thereby meeting this acceptance criterion (Table 3). Additionally, two out of three serum samples had 100% of dilution levels with a total %GCV < 50, which met the acceptance criterion (Table 3). However, one sample did not meet the acceptance criterion as only 71.4% (5 out of 7) of the dilution levels had total %GCV < 50 (Table 3).

**Figure 2:**
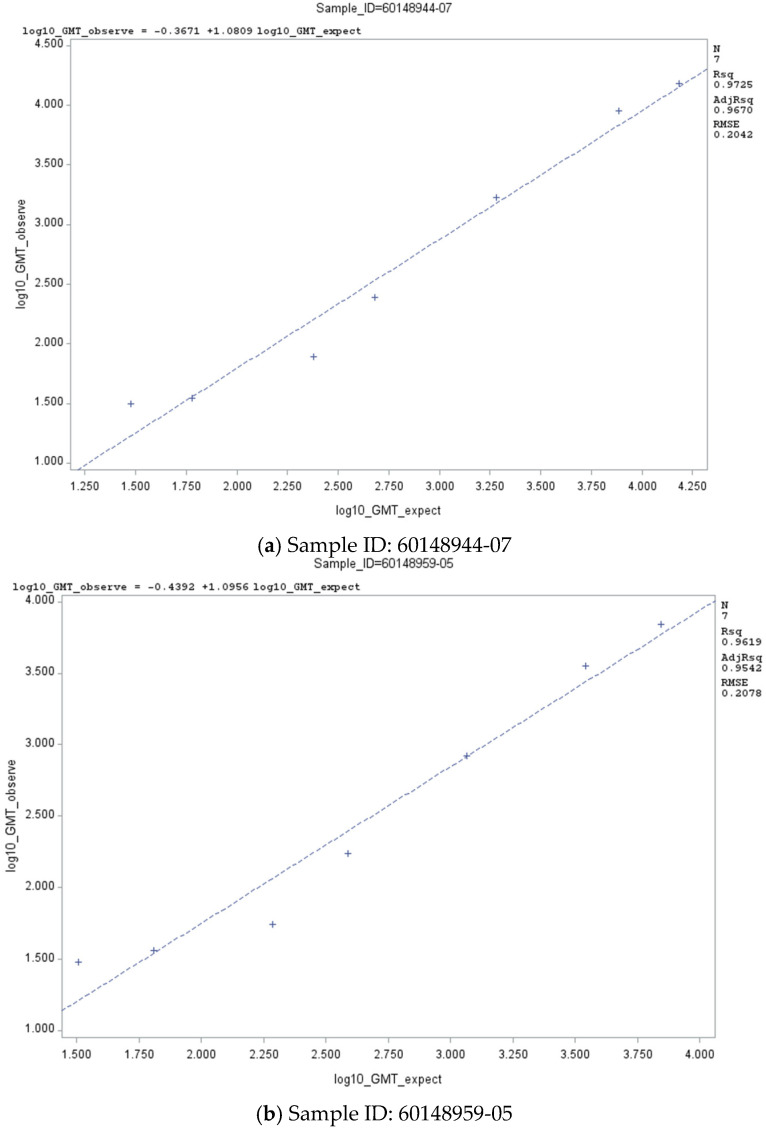
Linear regression plots of PNT GMT against Omicron JN.1 subvariant—specific matched (‘homologous’) JN.1 clinical sera. Abbreviations: GMT, geometric mean titer; ID, identification; PNT, pseudovirus neutralization titer.

A linearity analysis of the PNT assay was also performed using two clinically matched (‘homologous’) JN.1 serum samples with neutralizing antibodies against the Omicron JN.1 subvariant. Both samples met the targeted acceptance criteria for slope and *R*^2^ (Table 2). The linear regression plots are presented in Figure 2a,b. Five out of six of the dilution levels (83%) for each of the serum samples had % relative bias between −60 and 150, thereby meeting this acceptance criterion (Table 3). Additionally, 100% of the dilution levels of each of the serum samples had a total %GCV < 50, which met this acceptance criterion (Table 3).

**Figure 3:**
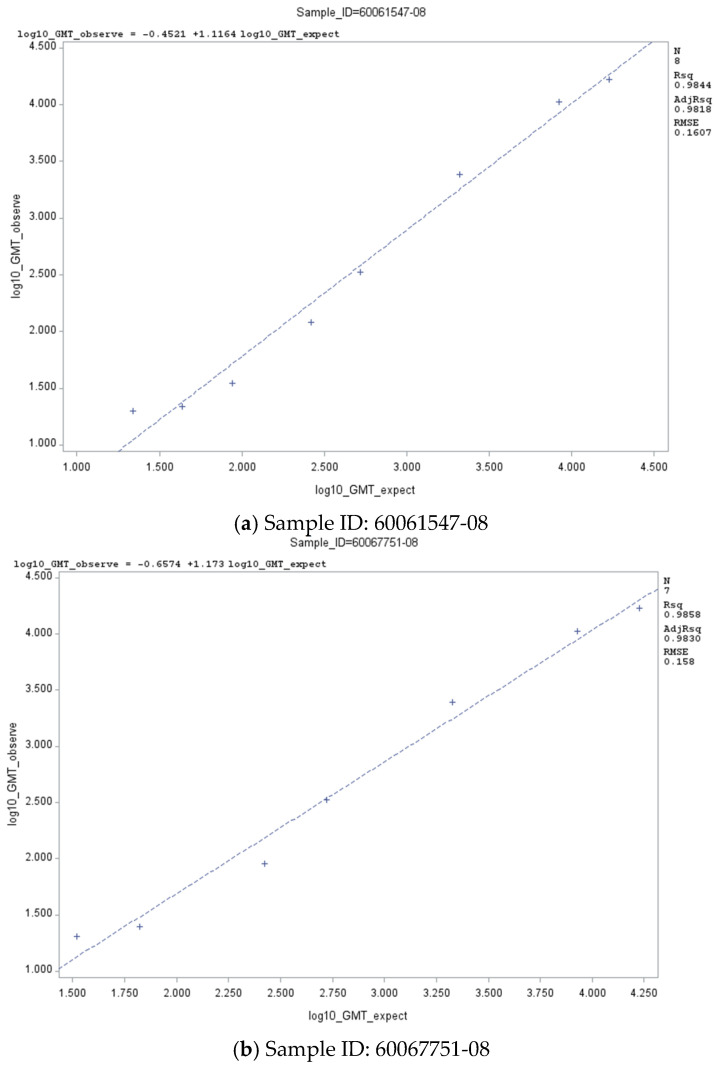
Linear regression plots of PNT GMT against Omicron LP.8.1 subvariant—non-matched (‘heterologous’) JN.1 clinical sera. Abbreviations: GMT, geometric mean titer; ID, identification; PNT, pseudovirus neutralization.

For the Omicron LP.8.1 subvariant, the linearity analysis of the PNT assay was performed using two non-matched (heterologous’) JN.1 serum samples with cross-reacting neutralizing antibodies against the Omicron LP.8.1 subvariant. Both samples met the targeted acceptance criteria for slope and *R*^2^ (Table 2). The linear regression plots are presented in Figure 3a,b. Both serum samples met the acceptance criterion for at least 80% dilution levels with a total %GCV < 50 (Table 3).

The linearity analysis of the PNT assay for the Omicron KP.2 and KP.3 subvariants, performed using two non-matched (‘heterologous’) sera with cross-reacting neutralizing antibodies against each of the Omicron KP.2 or KP.3 subvariants met the targeted acceptance criteria for slope and *R*^2^ for 4 out of 4 serum samples, except for the UCL for one sample of the Omicron KP.2 subvariant (Supplementary Table S2). The linear regression plots for the Omicron KP.2 subvariant samples are presented in Supplementary Figure S2a–d, and those for the Omicron KP.3 subvariant samples are presented in Supplementary Figure S2e–h. Additional evaluation of the PNT assay for the Omicron KP.2 and KP.3 subvariants showed that they also met acceptance criteria for GMT, % relative bias between −60 and 150, and total %GCV < 50 (Supplementary Table S3).

#### 3.1.3. Specificity

The results demonstrating the specificity of the SARS-CoV-2 PNT assay are presented in Supplementary Table S4. The human serum samples collected in the pre–COVID-19 era had no detectable titer against the Omicron JN.1, KP.2, KP.3, and LP.8.1 subvariants (<LLOQ). The LLOQ represents the lowest quantity of the analyte that can be accurately, precisely, and reproducibly quantitated in the assay. Furthermore, all samples from influenza and RSV clinical trial studies tested negative (<LLOQ) in the JN.1, KP.2, and KP.3 PNT assay while having very strong specific immune responses to influenza or RSV post-vaccination, demonstrating the specificity of the PNT assay.

### 3.2. Sensitivity and LLOQ and ULOQ

For the Omicron JN.1 subvariant, two samples were evaluated for precision and accuracy (% relative bias). GMTs of 30 and 15,312 were defined as the assay LLOQ and ULOQ, respectively. For the Omicron LP.8.1 subvariant, two samples were evaluated for precision and accuracy (% relative bias). GMTs of 35 and 16,775 were defined as the assay LLOQ and ULOQ, respectively. For KP.2 and KP.3, four samples each were assessed for precision and accuracy (% relative bias). GMTs of 35 for KP.2 and 23 for KP.3 were defined as assay LLOQ. The ULOQ was found to be 16,141 for KP.2 and 14,721 for KP.3.

### 3.3. Cross-Reactivity of 2019nCoV-311 Part 2 Serum Samples Against Omicron JN.1 Lineage Strains (KP.2, KP.3, KP.3.1.1, XEC, and LP.8.1 Subvariants)

The clinical utility of the PNT assay was assessed by profiling serum samples from the Novavax 2019nCoV-311 part 2 trial against the Omicron subvariants. ID50 GMT was highest against the Omicron JN.1 subvariant, while it was lowest against the Omicron KP.2 subvariant. ID50 GMTs against Omicron JN.1, KP.2, KP.3, KP.3.1.1, XEC, and LP.8.1 subvariants were 6708, 3094, 5924, 3732, 5404, and 3163, respectively. No significant differences (*p* > 0.5) were observed in neutralization antibody titers among the five Omicron subvariants tested (Supplementary Figure S1).

## 4. Discussion

This study describes the validation of the PNT assay for measuring neutralizing antibodies against SARS-CoV-2 Omicron subvariants, namely, JN.1, LP.8.1, KP.2, and KP.3 in human serum samples. The PNT assay exhibited acceptable precision, specificity, linearity, and sensitivity for neutralizing and/or cross-neutralizing antibodies against all four Omicron subvariants in human serum samples, underscoring its suitability for analyzing clinical samples from SARS-CoV-2 vaccine trials. Additionally, the clinical relevance of this assay was demonstrated in serum samples tested against the Omicron subvariants JN.1, KP.2, KP.3, KP.3.1.1, XEC, and LP.8.1. These results demonstrate potential cross-neutralization among the Omicron JN.1 subvariant and its descendants. Since non-matched sera are typically available early on and since the cross-reactivity of NAbs across strains of the same lineage—i.e., the JN.1 lineage in this case—is known, this approach validates the use of the PNT assay while avoiding the need to wait for specific matched sera.

As the COVID-19 continues to circulate globally, new variants of SARS-CoV-2, such as the Omicron subvariants, are constantly emerging with increased immune evasion ability [14]. These variants pose new challenges for current vaccines, necessitating modifications to vaccine antigen composition to enhance effectiveness against circulating strains and alleviate the impact on public health [15,16,17,18]. Since neutralizing antibodies are considered as correlates of protection (CoP) for vaccines, their quantification in COVID-19 cases facilitates the monitoring of viral infections and assesses the efficacy of existing and new vaccines [3,19,20].

Live virus-based neutralization assays are low-throughput and often limited by the risks associated with handling pathogenic viruses, the need for biosafety level (BSL)-3 laboratories, and time-consuming procedures [3,21]. By contrast, the PNT assay is a robust, high-throughput, and safer alternative that can be performed in a BSL-2 facility [3,4,5,22]. Cai et al. [5] reported the development and validation of a PNT assay for the SARS-CoV-2 ancestral strain (Wuhan) and the Omicron BA.5 and XBB.1.5 subvariants. The correlation analysis and Bland–Altman assay results confirmed the concordance between the PNT assay and the whole virus-based microneutralization assay, further validating the reliability of the PNT assay and indicating it as a safer alternative for determining neutralization titers [5]

The validation of the PNT assay confirms its reliability and reproducibility, supporting its use in clinical trials to determine vaccine immunogenicity in case of emerging and re-emerging variants [5,23]. In the present study, 87.5% (and 95%), 92.0%, 95.0%, and 92.5% of samples for Omicron JN.1, LP.8.1, KP.2, and KP.3 subvariants, respectively, met the precision criterion (overall assay %GCV ≤ 50% for ≥80% of positive samples) for measuring PNT titers in human serum samples using the PNT assay. The PNT assay was additionally accurate for all four Omicron subvariants as the % relative bias was within the expected range of −60 to 150 for almost or all dilution levels of the tested samples. The PNT was specific for the SARS-CoV-2 variants used in the validation experiments. Samples of sera that reflected strong and specific immune responses to their respective viruses such as seasonal influenza or RSV after vaccination showed no reactivity to the SARS-CoV-2 variants. Furthermore, the assay linearity was demonstrated for antisera dilution and LLOQ; ULOQ was established using high-titer sera where the sample’s %GCV was ≤50% and the sample fit was within the linear range.

Cross-reactivity against multiple variants is beneficial in light of the ongoing viral evolution and the emergence of escape variants [24]. In the present study, we assessed immune responses in human serum samples from the 2019nCoV-311 part 2 trial. The results demonstrated that the PNT assay was effective in measuring the neutralizing antibody activity against potentially immune-escaping Omicron subvariants JN.1, LP.8.1, KP.2, KP.3, KP.3.1.1, and XEC. No significant differences in neutralization antibody titers were observed among Omicron subvariants, highlighting its potential for immunoprofiling of new variants as they arise [4]. However, imprinting was observed as absolute titers were lower with the JN.1 vaccine compared to that for previous titers with Wuhan and earlier variants [1]. It is important to note that the PNT assay detects strain-specific neutralizing antibodies irrespective of the prior infection, vaccination, or hybrid immunity.

The PNT assay validated in the present study had some limitations. Here, we describe the validation of a SARS-CoV-2 PNT assay for the assessment and demonstration of neutralizing antibody responses elicited by COVID-19 vaccination. Although immune responses other than neutralizing antibodies were not evaluated, the Novavax SARS-CoV-2 protein-based vaccine has been shown to induce robust, durable, and cross-reactive CD4^+^ T-cell responses across multiple variants [25,26,27]. A modest increase in salivary IgA was observed following Novavax COVID-19 vaccination [28], consistent with findings reported for other intramuscular SARS-CoV-2 vaccines. In the PNT assay described here, the ULOQ was determined using high-titer serum samples that were available at the time of assay validation. The distribution and density of spike proteins on pseudoviruses may not accurately reflect their expression levels on natural viruses [4,5]. PNT assays mimic receptor interaction and cell entry but do not support productive infection of live viruses, highlighting the need for initial validation against live virus-based neutralization assays [29]. However, a significant correlation between PNT and a live virus assay has been demonstrated (Wuhan, other strains) to support the use of PNT as a CoP for the Wuhan strain [5]. Another limitation of this assay was that it did not detect antibodies that neutralize the binding of the SARS-CoV-2 spike protein to receptors other than angiotensin converting enzyme 2, the primary receptor for infection [30,31,32]. The role of additional candidate receptors in the pathogenesis of SARS-CoV-2, such as kringle containing transmembrane protein 1, a sialoglycoprotein receptor 1, and AXL are still unknown [33,34] and therefore their role cannot be denied and requires further investigation.

## 5. Conclusions

The data suggest that the validated PNT assay was precise, accurate, linear, and specific in measuring neutralization titers for SARS-CoV-2 Omicron subvariants, namely, JN.1, LP.8.1, KP.2, and KP.3 in human serum. This assay is suitable for the analysis of samples from SARS-CoV-2 vaccine clinical trials, enabling the rapid determination of vaccine immunogenicity against the circulating Omicron subvariants (JN.1 lineage). Future research aims to further confirm the correlation between the PNT assay results and live virus-based neutralization assays with the latest new strain lineages beyond JN.1. Additionally, efforts will be directed towards applying current PNT assays and adapting and optimizing the assay as needed for emerging SARS-CoV-2 variants.

## Figures and Tables

**Table 1:** Summary of overall precision validation PNT assay for Omicron JN.1 and LP.8.1 subvariants.

**Variants**	**Samples (N)**	**Intra-Assay** **%GCV**	**Inter-Assay** **%GCV**	**Total** **%GCV**
**JN.1^a^**	40	28.9	21.5	36.6
**JN.1^b^**	40	31.6	14.5	35.1
**LP.8.1**	40	36.0	13.1	38.6
		**Intra-Assay** **%GCV ≤ 50%**	**Inter-Assay** **%GCV ≤ 50%**	**Total** **%GCV ≤ 50%**
**JN.1^a^**	40	40 (100.0)	40 (100.0)	35 (87.5)
**JN.1^b^**	40	40 (100.0)	39 (98.0)	38 (95.0)
**LP.8.1**	40	39 (97.0)	40 (100.0)	37 (92.0)

Abbreviations: GCV, geometric coefficient of variation; PNT, pseudovirus neutralization. Note: Overall assay precision with JN.1 and LP.8.1 was calculated at the strain level by considering all 40 individual samples including positive and negative controls. Note: For JN.1^b^, 4 of 44 samples (2019nCov-315 study) tested < LLOQ with PNT GMT of 30.0. JN.1^a^ Original validation using ancestral Wuhan sera—2019nCoV-301 study samples (non-matched strain). JN.1^b^ Revalidation using JN.1 sera—2019nCoV-315 study samples (matched strain).

**Table 2:** Linear regression parameters of the PNT assay with Omicron JN.1 and LP.8.1 subvariants.

Variants	Sample IDs	Parameter	Estimate	95% LCL	95% UCL
**JN.1^a^**	NCN60488-01	Slope	1.091	0.957	1.225
		Intercept	−0.246	−0.590	0.098
		*R* ^2^	0.9922	N/A	
	NDE76198-02	Slope	1.041	0.927	1.156
		Intercept	−0.168	−0.456	0.120
		*R* ^2^	0.9938	N/A	
	HMN934977	Slope	1.058	0.947	1.168
		Intercept	−0.154	−0.435	0.127
		*R* ^2^	0.9944	N/A	
**JN.1^b^**	60148944-07	Slope	1.081	0.872	1.290
		Intercept	−0.367	−0.986	0.252
		*R* ^2^	0.9725	NA	
	60148959-05	Slope	1.096	0.845	1.346
		Intercept	−0.439	−1.14	0.258
		*R* ^2^	0.9619	NA	
**LP.8.1**	60061547-08	Slope	1.116	0.976	1.257
		Intercept	−0.452	−0.855	0.049
		*R* ^2^	0.9844	NA	
	600067751-08	Slope	1.173	1.011	1.335
		Intercept	−0.657	−1.14	−0.171
		*R* ^2^	0.9858	NA	

Abbreviations: LCL, lower confidence limit; NA, not available; PNT, pseudovirus neutralization; *R*^2^, coefficient of determination; UCL, upper confidence limit. JN.1^a^ Original validation using ancestral Wuhan sera—2019nCoV-301 study samples (not matched strain). JN.1^b^ Revalidation using JN.1 sera—2019nCoV-315 study samples (matched strain).

**Table 3:** Observed and expected GMT, % relative bias, and total %GCV evaluation of the PNT assay with Omicron JN.1 and LP.8.1 subvariants.

Sample	Dilution ^1^	N **	Overall Observed PNT GMT	Expected PNT GMT	% Relative Bias	Total % GCV
**JN.1^a^**						
**NCN60488-01**	1	12	2743.1	2743.1	0.0	46.1
	2	12	1811.0	1371.6	32.0	43.9
	6	12	503.5	457.2	10.1	50.9
	18	12	122.6	152.4	−19.5	34.0
	36	12	54.1	76.2	−28.9	49.5
	72	12	34.7	38.1	−8.9	36.7
	144	12	23.0	19.0	20.8	29.0
	288	12	20.0	9.5	110.0	0.0
**NDE76198-02**	1	12	2277.6	2277.6	0.0	60.5
	2	12	1086.8	1138.8	−4.6	52.2
	4	12	501.8	569.4	−11.9	31.7
	15	12	101.4	151.8	−33.2	38.9
	45	12	37.0	50.6	−27.0	36.7
	90	12	24.2	25.3	−4.5	34.1
	180	12	20.0	12.7	58.1	0.0
**HMN934977**	1	12	2278.1	2278.1	0.0	31.3
	2	12	1442.1	1139.1	26.6	41.0
	4	12	589.6	569.5	3.5	25.2
	12	12	153.1	189.8	−19.3	28.0
	36	12	52.5	63.3	−17.0	15.3
	72	12	30.4	31.6	−3.8	17.2
	144	12	21.4	15.8	35.4	13.5
**JN.1^b^**						
**60148944-07**	1	12	15,312.1	15,312.1	0.0	36.8
	2	12	8943.0	7656.0	16.8	41.1
	8	12	1695.2	1914.0	−11.4	38.5
	32	12	245.5	478.5	−48.7	42.8
	64	12	77.2	239.3	−67.7	22.1
	256	12	35.1	59.8	−41.3	37.1
	512	12	31.2	29.9 ^2^	4.3	13.0
	1024	12	30.0	15.0	100.6	0.0
**60148959-05**	1	12	6939.9	6939.9	0.0	42.9
	2	12	3581.7	3469.9	3.2	35.7
	6	12	841.9	1156.6	−27.2	30.1
	18	12	171.8	385.5	−55.4	42.2
	36	12	55.7	192.8	−71.1	19.9
	108	12	36.2	64.3	−43.7	29.9
	216	12	30.0	32.1	−6.6	0.0
	432	12	30.0	16.1	86.7	0.0
**LP.8.1**						
**60061547-08**	1	12	16,774.7	16,774.7	0.0	49.3
	2	12	10,585.4	8387.4	26.2	31.3
	8	12	2404.7	2096.8	14.7	48.4
	32	12	330.9	524.2	−36.9	44.6
	64	12	120.6	262.1	−54	52.6
	192	12	35.2	87.4	−59.7	42.4
	384	12	21.8	43.7	−50.0	25.1
	768	12	20.0	21.8	−8.4	0.0
**60067751-08**	1	12	16,932.4	16,932.4	0.0	47.2
	2	12	10,583.2	8466.2	25.0	27.3
	8	12	2472.5	2116.6	16.8	37.9
	32	12	333.1	529.1	−37.0	54.7
	64	12	90.8	264.6	−65.7	40.5
	256	12	24.5	66.1	−62.9	39.6
	512	12	20.2	33.1	−39.0	4.6
	1024	12	20.2	16.5	22.4	6.1

Note: Data points with the expected titer at and above 20 were used for plotting the linear regression curve. A PNT titer of <20 (sample minimum required dilution prior to LLOQ determination) was defined as 20 for calculation purposes. For JN.1^b^, data points with the expected titer at and above LLOQ of 30 were used for plotting the linear regression curve. A PNT titer of <30 was defined as 30 for calculation purposes. ^1^ Dilution factors that were applied to dilute different serum samples to below-assay LLOQ in linearity experiments. Dilution with expected GMT < 20 (MRD) or <30 (LLOQ) was excluded from linearity fitting. ^2^ Rounded to 30 and included for plotting the linearity regression curve. ** Number of GMT values used for calculation. Abbreviations: GCV, geometric coefficient of variation; GMT, geometric mean titer; PNT, pseudovirus neutralization. JN.1^a^ Original validation using ancestral Wuhan sera—2019nCoV-301 study samples (non-matched strain). JN.1^b^ Revalidation using JN.1 sera—2019nCoV-315 study samples (matched strain).

## Data Availability

Requests for the data presented in this study will be considered by the corresponding author. The data are not publicly available because of proprietary subject and sample information.

## Supplementary Materials

The following supporting information can be downloaded at: https://www.mdpi.com/article/10.3390/microorganisms14051042/s1, Table S1; Precision validation PNT assay results for Omicron KP.2 and KP.3 subvariants; Table S2: Linear regression parameters of the PNT assay with Omicron KP.2 and KP.3 subvariants; Table S3: Observed and expected GMT, % relative bias, and total %GCV evaluation of the PNT assay for Omicron KP.2 and KP.3 subvariants; Table S4: Specificity results of the PNT assay in human sera and with Omicron JN.1, KP.2, KP.3, and LP.8.1 subvariants in the RSV F protein- and influenza-vaccinated clinical sera pairs; Table S5: Omicron JN.1, KP.2, and KP.3 subvariant human serum samples tested in the PNT assay; Figure S1: Cross-reactivity of 2019nCoV-311 part 2 serum samples (Wuhan and BA5 vaccinated) against Omicron JN.1, KP.2, KP.3, KP.3.1.1, XEC, and LP.8.1 subvariants; Figure S2: Linear regression plots of PNT GMTs against Omicron KP.2 and KP.3 subvariants—non-matched (‘heterologous’) JN.1 clinical sera.
