# Supplementary material for: Validation of a Pseudovirus Neutralization Assay for Severe Acute Respiratory Syndrome Coronavirus 2 Omicron JN.1 and LP.8.1 Subvariant Lineage Strains with Homologous and Heterologous Matched Sera in Clinically Relevant Samples
Source: Microorganisms. 2026 May 5;14(5):1042. doi: 10.3390/microorganisms14051042 (PMC13209548; doi:10.3390/microorganisms14051042)
Supplement: Supplementary file 1 [file microorganisms-14-01042-s001.zip › microorganisms-4265509-supplementary.pdf]

SUPPLEMENT:

Table S1. Precision validation PNT assay results for Omicron KP.2 and KP.3 subvariants.

Variants	Samples (N)	Intra-assay %GCV	Inter-assay %GCV	Total %GCV
**KP.2**	40	29.1	10.0	30.9
**KP.3**	40	27.7	1.8	27.8
		**Intra-assay %GCV ≤50%**	**Inter-assay %GCV ≤50%**	**Total %GCV ≤50%**
**KP.2**	40	39 (97.5)	40 (100.0)	38 (95.0)
**KP.3**	40	39 (97.5)	40 (100.0)	37 (92.5)

Abbreviations: GCV, geometric coefficient of variation; PNT, pseudovirus neutralization.

Table S2. Linear regression parameters of the PNT assay with Omicron KP.2 and KP.3 subvariants.

Variants	Sample IDs	Parameter	Estimate	95% LCL	95% UCL
**KP.2**	60061577-07	Slope	1.171	0.914	1.429
		Intercept	−0.502	−1.210	0.205
		R^2	0.9755	N/A	
	60067751-07	Slope	1.213	0.964	1.462
		Intercept	−0.795	−1.560	−0.033
		R^2	0.9787`	N/A	
	60065748-07	Slope	1.199	1.000	1.397
		Intercept	−0.648	−1.230	−0.068
		R^2	0.9796	NA	
	60053573-07	Slope	1.129	0.997	1.261
		Intercept	−0.475	−0.862	−0.088
		R^2	0.9864	NA	
**KP.3**	60061577-07	Slope	1.174	0.963	1.386
		Intercept	−0.626	−1.22	−0.029
		R^2	0.9834	NA	
	60067751-07	Slope	1.154	1.027	1.281
		Intercept	−0.621	−1.01	−0.234
		R^2	0.9938	NA	
	60065748-07	Slope	1.075	0.875	1.275
		Intercept	−0.097	−0.639	0.444
		R^2	0.9746	NA	
	60053573-07	Slope	1.097	0.966	1.228
		Intercept	−0.288	−0.666	0.09
		R^2	0.9859	NA	

Abbreviations: LCL, lower confidence limit; NA, not available; PNT, pseudovirus neutralization; R2, coefficient of determination; UCL, upper confidence limit.

Table S3. Observed and expected GMT, % relative bias, and total %GCV evaluation of the PNT assay for Omicron KP.2 and KP.3 subvariants

Sample	Dilution	N[**]	Overall observed PNT GMT	Expected PNT GMT	% Relative bias	Total % GCV[‖]
**KP.2**						
**60061577-07**[‡]	1	12	6322.9	6322.9	0.0	47.5
	2	12	5333.1	3161.5	68.7	45.5
	8	12	1198.8	790.4	51.7	40.1
	32	12	106.0	197.6	−46.4	59.8
	128	12	20.0	49.4	−59.5	0.0
	256	12	20.0	24.7	−19.0	0.0
	512	12	20.0	12.3	62.0	0.0
	1024	12	20.0	6.2	223.9	0.0
**60067751-07**[¶]	1	12	14,207.5	14,207.5	0.0	48.4
	2	12	8749.4	7103.7	23.2	43.4
	8	12	1866.6	1775.9	5.1	38.4
	32	12	274.8	444.0	−38.1	34.4
	128	12	23.4	111.0	−78.9	21.1
	384	12	20.0	37.0	−45.9	0.0
	768	12	20.0	18.5	8.1	0.0
	1536	12	20.0	9.2	116.2	0.0
**60065748-07**[$]	1	6	10,859.2	10,859.2	0.0	24.2
	2	6	10,476.0	5429.6	92.9	24.7
	6	6	2641.0	1809.9	45.9	36.7
	18	6	355.4	603.3	−41.1	18.9
	54	6	81.7	201.1	−59.4	13.7
	162	6	35.2	67.0	−47.5	37.8
	324	6	20.3	33.5	−39.4	3.9
	648	6	20.0	16.8	19.3	0.0
**60053573-07**[§]	1	6	16,141.2	16,141.2	0.0	36.8
	2	6	11,572.3	8070.6	43.4	45.1
	6	6	3435.1	2690.2	27.7	49.5
	18	6	572.2	896.7	−36.2	32.9
	54	6	143.5	298.9	−52	13.1
	162	6	48.0	99.6	−51.8	28.4
	324	6	25.2	49.8	−49.5	17.9
	648	6	20.5	24.9	−17.8	5.7
**KP.3**						
**60061577-07**[‡]	1	12	7641.8	7641.8	0.0	51.4
	2	12	4592.4	3820.9	20.2	25.4
	8	12	937.9	955.2	−1.8	26.1
	32	12	100.1	238.8	−58.1	41.9
	128	12	20.0	59.7	−66.5	1.0
	256	12	20.0	29.9	−33.0	0.0
	512	12	20.0	14.9	34	0.0
	1024	12	20.0	7.5	168	0.0
**60067751-07**[¶]	1	12	14,001.3	14,001.3	0.0	35.6
	2	12	7103.3	7000.7	1.5	21.2
	8	12	1479.3	1750.2	−15.5	24.9
	32	12	236.7	437.5	−45.9	41.6

	128	12	38.7	109.4	−64.6	56.8
	384	12	20.2	36.5	−44.5	4.2
	768	12	20.0	18.2	9.7	0.0
	1536	12	20.0	9.1	119.4	0.0
**60065748-07**[$]	1	6	6596.7	6596.7	0	33.4
	2	6	7017.4	3298.3	112.8	30.6
	6	6	2417.8	1099.4	119.9	47.3
	18	6	376.3	366.5	2.7	40.2
	54	6	86.2	122.2	−29.4	30.5
	162	6	49.0	40.7	20.3	30.1
	324	6	22.9	20.4	12.7	7.6
	648	6	20.0	10.2	96.5	0.0
**60053573-07**[§]	1	6	14,720.7	14,720.7	0.0	31.7
	2	6	11,771	7360.3	59.9	22.5
	6	6	4203.8	2453.4	71.3	47.8
	18	6	697.1	817.8	−14.8	39.3
	54	6	151.9	272.6	−44.3	8.4
	162	6	60.4	90.9	−33.6	28.1
	324	6	38.3	45.4	−15.7	31
	648	6	20.2	22.7	-11.2	2.0

Note: Data points with the expected titer at and above 20 were used for plotting the linear regression curve. PNT titer of <20 was defined as 20 for calculation purposes.

*Dilution factors ranging from 1/1 to 1/72 were used in linearity regression summary and plot. Dilution with expected GMT <20 was excluded from linearity fitting.

‡Dilution factors ranging from 1/1 to 1/256 were used in linearity regression summary and plot. Dilution with expected GMT <20 was excluded from linearity fitting.

¶Dilution factors ranging from 1/1 to 1/384 were used in linearity regression summary and plot. Dilution with expected GMT <20 was excluded from linearity fitting.

$Dilution factors ranging from 1/1 to 1/324 were used in linearity regression summary and plot. Dilution with expected GMT <20 was excluded from linearity fitting.

§Dilution factors ranging from 1/1 to 1/648 were used in linearity regression summary and plot. Dilution with expected GMT <20 was excluded from linearity fitting.

**Number of GMT values used for calculation.

||Variance component analysis was used for samples 60061577-07 and 60067751-07 precision analysis as part of the precision runs but not used for samples 60061548-07 and 60053573-07 precision analysis (only total %GCV was calculated).

Abbreviations: GCV, geometric coefficient of variation; GMT, geometric mean titer; PNT, pseudovirus neutralization.

Table S4. Specificity results of the PNT assay in human sera and with Omicron JN.1, KP.2, KP.3, and LP.8.1 subvariants in the RSV F protein- and influenza-vaccinated clinical sera pairs

JN.1		LP.8.1		KP.2		KP.3	
**Pre-COVID-19 Normal Human Sera**	**PNT Titer**	**Pre-COVID-19 Normal Human Sera**	**PNT Titer**	**Pre-COVID-19 Normal Human Sera**	**PNT Titer**	**Pre-COVID-19 Normal Human Sera**	**PNT Titer**
BRH1452647	<LLOQ	BRH1452647	<LLOQ	BRH1452647	<LLOQ	BRH1452647	<LLOQ
BRH1452659	<LLOQ	BRH1452659	<LLOQ	BRH1452659	<LLOQ	BRH1452659	<LLOQ
LS 24 00429A	<LLOQ	LS 24 00429A	<LLOQ	LS 24 00429A	<LLOQ	LS 24 00429A	<LLOQ
LS 88 35276A	<LLOQ	LS 88 35276A	<LLOQ	LS 88 35276A	<LLOQ	LS 88 35276A	<LLOQ
BRH1224485	<LLOQ	BRH1224485	<LLOQ	BRH1224485	<LLOQ	BRH1224485	<LLOQ
BRH1468488	<LLOQ	BRH1468488	<LLOQ	BRH1468488	<LLOQ	BRH1468488	<LLOQ
LS 24 00120A	<LLOQ	LS 24 00120A	<LLOQ	LS 24 00120A	<LLOQ	LS 24 00120A	<LLOQ
LS 24 00122A	<LLOQ	LS 24 00122A	<LLOQ	LS 24 00122A	<LLOQ	LS 24 00122A	<LLOQ

JN.1, KP.2, and KP.3, and LP.8.1 PNT titer (ID_{50}) in RSV F protein-vaccinated sera taken pre- and post-RSV F vaccination

Participant ID	Anti-RSV F Protein Antibody			JN.1, KP.2, KP.3, and LP.8.1 PNT Titer		
	**Pre-RSV F Vaccination (Day 0)**	**Post-RSV F Vaccination (Day 14)**	**Post-/Pre-Vaccination Ratio**	**Pre-RSV F Vaccination (Day 0)**	**Post-RSV F Vaccination (Day 14)**	**Post-/Pre-Vaccination Ratio**
US031-3011	509	43,767	86	<LLOQ	<LLOQ	1
US032-3028	474	20,188	43	<LLOQ	<LLOQ	1
US089-2003	483	9721	20	<LLOQ	<LLOQ	1
US123-3072	632	10,958	17	<LLOQ	<LLOQ	1
US125-3002	772	15,869	21	<LLOQ	<LLOQ	1

JN.1, KP.2, KP.3, and LP.8.1 PNT titer (ID_{50}) in influenza-vaccinated sera taken pre- and post-influenza vaccination						
	Influenza HAI Titer (A/Kansas/14/2017) (H3N2)			JN.1, KP.2, KP.3, and LP.8.1 PNT Titer		
Participant ID	Pre-Influenza Vaccination (Day 0)	Post-Influenza Vaccination (Day 28)	Post-/Pre-Vaccination Ratio	Pre-Influenza Vaccination (Day 0)	Post-Influenza Vaccination (Day 28)	Post-/Pre-Vaccination Ratio
US012-1139	10	640	64	<LLOQ	<LLOQ	1
US013-1050	20	1280	64	<LLOQ	<LLOQ	1
US025-1082	10	1280	128	<LLOQ	<LLOQ	1
US032-1003	20	2560	128	<LLOQ	<LLOQ	1
US025-1035	20	1280	64	<LLOQ	<LLOQ	1

Note: LLOQ was 30 for JN.1, 35 for KP.2, 23 for KP.3, and 35 for LP.8.1.

Abbreviations: COVID-19, coronavirus disease 2019; HAI, hemagglutination inhibition; LLOQ, lower limit of quantitation; PC, positive control; PNT, pseudovirus neutralization titer; RSV, respiratory syncytial virus.

Table S5. Omicron JN.1, KP.2, and KP.3 subvariant human serum samples tested in the PNT assay

Group	Sample ID	Source
**JN.1**		
In-House	12-2024-041	Prepared in house by 1:9 dilution of 35-2024-074 with human serum 35-2023-075 (in house prepared)
BioIVT	HMN865537	BioIVT
	35-2023-074	Prepared in house by 1:4 dilution of HMN865537 with human serum HMN8655541 (BioIVT)
	HMN865529	BioIVT
	NCN60488-01 (1:2)	Prepared in house by 1:2 dilution of NCN60488-01 with human serum HMN865541 (BioIVT)
	NCN60488-01 (1:6)	Prepared in house by 1:6 dilution of NCN60488-01 with human serum HMN865541 (BioIVT)
	NCN60488-01 (1:18)	Prepared in house by 1:18 dilution of NCN60488-01 with human serum HMN865541 (BioIVT)
	NCN60488-01 (1:36)	Prepared in house by 1:36 dilution of NCN60488-01 with human serum HMN865541 (BioIVT)
	NCN60488-01 (1:72)	Prepared in house by 1:72 dilution of NCN60488-01 with human serum HMN865541 (BioIVT)
	NCN60488-01 (1:144)	Prepared in house by 1:144 dilution of NCN60488-01 with human serum HMN865541 (BioIVT)
	NCN60488-01 (1:288)	Prepared in house by 1:288 dilution of NCN60488-01 with human serum HMN865541 (BioIVT)
	NDE76198-02 (1:2)	Prepared in house by 1:2 dilution of NDE76198-02 with human serum HMN865541 (BioIVT)
	NDE76198-02 (1:4)	Prepared in house by 1:4 dilution of NDE76198-02 with human serum HMN865541 (BioIVT)
	NDE76198-02 (1:15)	Prepared in house by 1:15 dilution of NDE76198-02 with human serum HMN865541 (BioIVT)
	NDE76198-02 (1:45)	Prepared in house by 1:45 dilution of NDE76198-02 with human serum HMN865541 (BioIVT)
	NDE76198-02 (1:90)	Prepared in house by 1:90 dilution of NDE76198-02 with human serum HMN865541 (BioIVT)
	NDE76198-02 (1:180)	Prepared in house by 1:180 dilution of NDE76198-02 with human serum HMN865541 (BioIVT)
	HMN865534	BioIVT
	HMN865536	BioIVT
	HMN865541	BioIVT
	HMN865545	BioIVT
	HMN865550	BioIVT
	HMN935018	BioIVT
	BRH1452647	BioIVT
	BRH1452659	BioIVT, screened HAI positive for at least 1 influenza strain
	BRH1224485	BioIVT, screened HAI negative for at least 1 influenza strain
	BRH1468488	BioIVT, screened HAI positive for at least 1 influenza strain
	HMN934977	BioIVT
	HMN934977 (1:2)	Prepared in house by 1:2 dilution of HMN934977 with human serum HMN865541 (BioIVT)

Group	Sample ID	Source
	HMN934977 (1:4)	Prepared in house by 1:4 dilution of HMN934977 with human serum HMN865541 (BioIVT)
	HMN934977 (1:2)	Prepared in house by 1:2 dilution of HMN934977 with human serum HMN865541 (BioIVT)
	HMN934977 (1:12)	Prepared in house by 1:12 dilution of HMN934977 with human serum HMN865541 (BioIVT)
	HMN934977 (1:36)	Prepared in house by 1:36 dilution of HMN934977 with human serum HMN865541 (BioIVT)
	HMN934977 (1:72)	Prepared in house by 1:72 dilution of HMN934977 with human serum HMN865541 (BioIVT)
	HMN934977 (1:144)	Prepared in house by 1:144 dilution of HMN934977 with human serum HMN865541 (BioIVT)
2019nCoV-301 Phase 3 Trial	NCN60488-01	Novavax COVID-19 vaccine phase 3 trial, 2019nCoV-301
	NDE76198-02	Novavax COVID-19 vaccine phase 3 trial, 2019nCoV-301
	NCH61391-01	Novavax COVID-19 vaccine phase 3 trial, 2019nCoV-301
	NCO50768-01	Novavax COVID-19 vaccine phase 3 trial, 2019nCoV-301
	NCH87195-01	Novavax COVID-19 vaccine phase 3 trial, 2019nCoV-301
	NCN85206-04	Novavax COVID-19 vaccine phase 3 trial, 2019nCoV-301
	NCI96173-13	Novavax COVID-19 vaccine phase 3 trial, 2019nCoV-301
	NCF20555-13	Novavax COVID-19 vaccine phase 3 trial, 2019nCoV-301
	NCN37273-01	Novavax COVID-19 vaccine phase 3 trial, 2019nCoV-301
	NCK70411-02	Novavax COVID-19 vaccine phase 3 trial, 2019nCoV-301
	NCG83119-01	Novavax COVID-19 vaccine phase 3 trial, 2019nCoV-301
	NBZ54499-01	Novavax COVID-19 vaccine phase 3 trial, 2019nCoV-301
	NCK70061-01	Novavax COVID-19 vaccine phase 3 trial, 2019nCoV-301
	NCP52304-01	Novavax COVID-19 vaccine phase 3 trial, 2019nCoV-301
	NCH99029-01	Novavax COVID-19 vaccine phase 3 trial, 2019nCoV-301
	NCN03843-13	Novavax COVID-19 vaccine phase 3 trial, 2019nCoV-301
	NCP07857-01	Novavax COVID-19 vaccine phase 3 trial, 2019nCoV-301
qNIV-E-301 Phase 3 Trial	10807371-03	Novavax influenza vaccine phase 3 trial, qNIV-E-301
	10807123-03	Novavax influenza vaccine phase 3 trial, qNIV-E-301
	10802296-03	Novavax influenza vaccine phase 3 trial, qNIV-E-301
	10802350-03	Novavax influenza vaccine phase 3 trial, qNIV-E-301
	10803093-04	Novavax influenza vaccine phase 3 trial, qNIV-E-301
	10807983-03	Novavax influenza vaccine phase 3 trial, qNIV-E-301
	10803811-03	Novavax influenza vaccine phase 3 trial, qNIV-E-301
	10809567-03	Novavax influenza vaccine phase 3 trial, qNIV-E-301
	10803108-03	Novavax influenza vaccine phase 3 trial, qNIV-E-301
	10808130-03	Novavax influenza vaccine phase 3 trial, qNIV-E-301
RSV-M-301 Phase 3 Trial	10472223-04	Novavax RSV vaccine phase 3 trial, RSV-M-301
	10472214-04	Novavax RSV vaccine phase 3 trial, RSV-M-301
	10514078-04	Novavax RSV vaccine phase 3 trial, RSV-M-301
	10342270-07	Novavax RSV vaccine phase 3 trial, RSV-M-301
	10342305-06	Novavax RSV vaccine phase 3 trial, RSV-M-301
	10509341-04	Novavax RSV vaccine phase 3 trial, RSV-M-301
	10513556-04	Novavax RSV vaccine phase 3 trial, RSV-M-301
	10343569-08	Novavax RSV vaccine phase 3 trial, RSV-M-301
	10452779-04	Novavax RSV vaccine phase 3 trial, RSV-M-301

Group	Sample ID	Source
Biological specialty Co	LS 24 00429A	Biological specialty Co., reported receipt of influenza vaccine within 1 year of the date received
	LS 88 35276A	Biological specialty Co., reported receipt of influenza vaccine within 1 year of the date received
	LS 24 00120A	Biological specialty Co., reported receipt of influenza vaccine within 1 year of the date received
	LS 24 00122A	Biological specialty Co., reported receipt of influenza vaccine within 1 year of the date received
**KP.2/KP.3**		
BioIVT	12-2024-425	Pool equal volume of 60066217-11, 60057263-11, 60057266-11, 60056800-11 and 60065746-08 from study 2019nCoV-311 part 2, then 1:5 diluted with human serum HMN934977 from BioIVT
	12-2024-426	In house prepared by diluting PC1 (12-2024-425) 1:5 with human serum HMN865529 from BioIVT
	12-2024-427	In house prepared by diluting PC1 (12-2024-425) 1:24 with human serum HMN865529 from BioIVT
	HMN865529	BioIVT
	60061577-07 (1:2)	Prepared in house by 1:2 dilution of 60061577-07 with human serum HMN865529 (BioIVT)
	60061577-07 (1:8)	Prepared in house by 1:8 dilution of 60061577-07 with human serum HMN865529 (BioIVT)
	60061577-07 (1:32)	Prepared in house by 1:32 dilution of 60061577-07 with human serum HMN865529 (BioIVT)
	60061577-07 (1:128)	Prepared in house by 1:128 dilution of 60061577-07 with human serum HMN865529 (BioIVT)
	60061577-07 (1:256)	Prepared in house by 1:256 dilution of 60061577-07 with human serum HMN865529 (BioIVT)
	60061577-07 (1:512)	Prepared in house by 1:512 dilution of 60061577-07 with human serum HMN865529 (BioIVT)
	60061577-07 (1:1024)	Prepared in house by 1:1024 dilution of 60061577-07 with human serum HMN865529 (BioIVT)
	60067751-07 (1:2)	Prepared in house by 1:2 dilution of 60067751-07 with human serum HMN865529 (BioIVT)
	60067751-07 (1:8)	Prepared in house by 1:8 dilution of 60067751-07 with human serum HMN865529 (BioIVT)
	60067751-07 (1:32)	Prepared in house by 1:32 dilution of 60067751-07 with human serum HMN865529 (BioIVT)
	60067751-07 (1:128)	Prepared in house by 1:128 dilution of 60067751-07 with human serum HMN865529 (BioIVT)
	60067751-07 (1:384)	Prepared in house by 1:384 dilution of 60067751-07 with human serum HMN865529 (BioIVT)
	60067751-07 (1:768)	Prepared in house by 1:768 dilution of 60067751-07 with human serum HMN865529 (BioIVT)
	60067751-07 (1:1536)	Prepared in house by 1:1536 dilution of 60067751-07 with human serum HMN865529 (BioIVT)
	BRH1452647	BioIVT
	BRH1452659	BioIVT, screened HAI positive for at least 1 influenza strain
	BRH1224485	BioIVT, screened HAI negative for at least 1 influenza strain
	BRH1468488	BioIVT, screened HAI positive for at least 1 influenza strain

Group	Sample ID	Source
	60065748-07 (1:2)	Prepared in house by 1:2 dilution of 60065748-07 with human serum HMN865541 (BioIVT)
	60065748-07 (1:6)	Prepared in house by 1:6 dilution of 60065748-07 with human serum HMN865541 (BioIVT)
	60065748-07 (1:18)	Prepared in house by 1:18 dilution of 60065748-07 with human serum HMN865541 (BioIVT)
	60065748-07 (1:54)	Prepared in house by 1:54 dilution of 60065748-07 with human serum HMN865541 (BioIVT)
	60065748-07 (1:162)	Prepared in house by 1:162 dilution of 60065748-07 with human serum HMN865541 (BioIVT)
	60065748-07 (1:324)	Prepared in house by 1:324 dilution of 60065748-07 with human serum HMN865541 (BioIVT)
	60065748-07 (1:648)	Prepared in house by 1:648 dilution of 60065748-07 with human serum HMN865541 (BioIVT)
	60053573-07	Novavax COVID-19 vaccine phase 3 trial, 2019nCoV-311 part 2
	60053573-07 (1:2)	Prepared in house by 1:2 dilution of 60053573-07 with human serum HMN865541 (BioIVT)
	60053573-07 (1:6)	Prepared in house by 1:6 dilution of 60053573-07 with human serum HMN865541 (BioIVT)
	60053573-07 (1:18)	Prepared in house by 1:18 dilution of 60053573-07 with human serum HMN865541 (BioIVT)
	60053573-07 1:54	Prepared in house by 1:54 dilution of 60053573-07 with human serum HMN865541 (BioIVT)
	60053573-07 (1:162)	Prepared in house by 1:162 dilution of 60053573-07 with human serum HMN865541 (BioIVT)
	60053573-07 (1:324)	Prepared in house by 1:324 dilution of 60053573-07 with human serum HMN865541 (BioIVT)
	60053573-07 (1:648)	Prepared in house by 1:648 dilution of 60053573-07 with human serum HMN865541 (BioIVT)
2019nCoV-311 part 2 Phase 3 Trial	60067751-07	Novavax COVID-19 vaccine phase 3 trial, 2019nCoV-311 part 2
	60068324-07	Novavax COVID-19 vaccine phase 3 trial, 2019nCoV-311 part 2
	60060753-07	Novavax COVID-19 vaccine phase 3 trial, 2019nCoV-311 part 2
	60060751-07	Novavax COVID-19 vaccine phase 3 trial, 2019nCoV-311 part 2
	60064114-08	Novavax COVID-19 vaccine phase 3 trial, 2019nCoV-311 part 2
	60068572-11	Novavax COVID-19 vaccine phase 3 trial, 2019nCoV-311 part 2
	60069432-07	Novavax COVID-19 vaccine phase 3 trial, 2019nCoV-311 part 2
	60060772-11	Novavax COVID-19 vaccine phase 3 trial, 2019nCoV-311 part 2
	60069258-07	Novavax COVID-19 vaccine phase 3 trial, 2019nCoV-311 part 2
	60053900-08	Novavax COVID-19 vaccine phase 3 trial, 2019nCoV-311 part 2
	60060558-11	Novavax COVID-19 vaccine phase 3 trial, 2019nCoV-311 part 2
	60067127-11	Novavax COVID-19 vaccine phase 3 trial, 2019nCoV-311 part 2
	60070861-08	Novavax COVID-19 vaccine phase 3 trial, 2019nCoV-311 part 2
	60060547-07	Novavax COVID-19 vaccine phase 3 trial, 2019nCoV-311 part 2
	60053900-08 (KP.2) 60065992-07 (KP.3)	Novavax COVID-19 vaccine phase 3 trial, 2019nCoV-311 part 2
	60054391-07	Novavax COVID-19 vaccine phase 3 trial, 2019nCoV-311 part 2
	60064181-07	Novavax COVID-19 vaccine phase 3 trial, 2019nCoV-311 part 2

Group	Sample ID	Source
	60064619-08	Novavax COVID-19 vaccine phase 3 trial, 2019nCoV-311 part 2
	60052996-11	Novavax COVID-19 vaccine phase 3 trial, 2019nCoV-311 part 2
	60054605-08	Novavax COVID-19 vaccine phase 3 trial, 2019nCoV-311 part 2
	60059863-07	Novavax COVID-19 vaccine phase 3 trial, 2019nCoV-311 part 2
	60061577-07	Novavax COVID-19 vaccine phase 3 trial, 2019nCoV-311 part 2
	60065748-07	Novavax COVID-19 vaccine phase 3 trial, 2019nCoV-311 part 2
qNIV-E-301 Phase 3 trial	10807371-03	Novavax influenza vaccine phase 3 trial, qNIV-E-301
	10807123-03	Novavax influenza vaccine phase 3 trial, qNIV-E-301
	10802296-03	Novavax influenza vaccine phase 3 trial, qNIV-E-301
	10802350-03	Novavax influenza vaccine phase 3 trial, qNIV-E-301
	10803093-04	Novavax influenza vaccine phase 3 trial, qNIV-E-301
	10807983-03	Novavax influenza vaccine phase 3 trial, qNIV-E-301
	10803811-03	Novavax influenza vaccine phase 3 trial, qNIV-E-301
	10809567-03	Novavax influenza vaccine phase 3 trial, qNIV-E-301
	10803108-03	Novavax influenza vaccine phase 3 trial, qNIV-E-301
	10808130-03	Novavax influenza vaccine phase 3 trial, qNIV-E-301
RSV-M-301 Phase 3 Trial	10472223-04	Novavax RSV vaccine phase 3 trial, RSV-M-301
	10472214-04	Novavax RSV vaccine phase 3 trial, RSV-M-301
	10475059-04	Novavax RSV vaccine phase 3 trial, RSV-M-301
	10514078-04	Novavax RSV vaccine phase 3 trial, RSV-M-301
	10342270-07	Novavax RSV vaccine phase 3 trial, RSV-M-301
	10342305-06	Novavax RSV vaccine phase 3 trial, RSV-M-301
	10509341-04	Novavax RSV vaccine phase 3 trial, RSV-M-301
	10513556-04	Novavax RSV vaccine phase 3 trial, RSV-M-301
	10343569-08	Novavax RSV vaccine phase 3 trial, RSV-M-301
	10452779-04	Novavax RSV vaccine phase 3 trial, RSV-M-301
Biological specialty Co	LS 24 00429A	Biological specialty Co., reported receipt of influenza vaccine within 1 year of the date received
	LS 88 35276A	Biological specialty Co., reported receipt of influenzas vaccine within 1 year of the date received
	LS 24 00120A	Biological specialty Co., reported receipt of influenza vaccine within 1 year of the date received
	LS 24 00122A	Biological specialty Co., reported receipt of influenza vaccine within 1 year of the date received

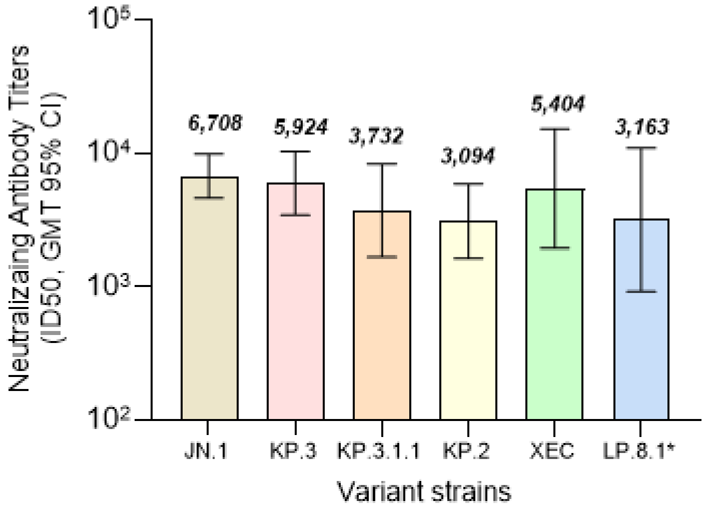

Figure S1. Cross-reactivity of 2019nCoV-311 part 2 serum samples (Wuhan and BA5 vaccinated) against Omicron JN.1, KP.2, KP.3, KP.3.1.1, XEC, and LP.8.1 sub-variants. *N = 11. Abbreviations: CI, confidence interval; GMT, geometric mean titer; ID50, 50% inhibitory dilution.

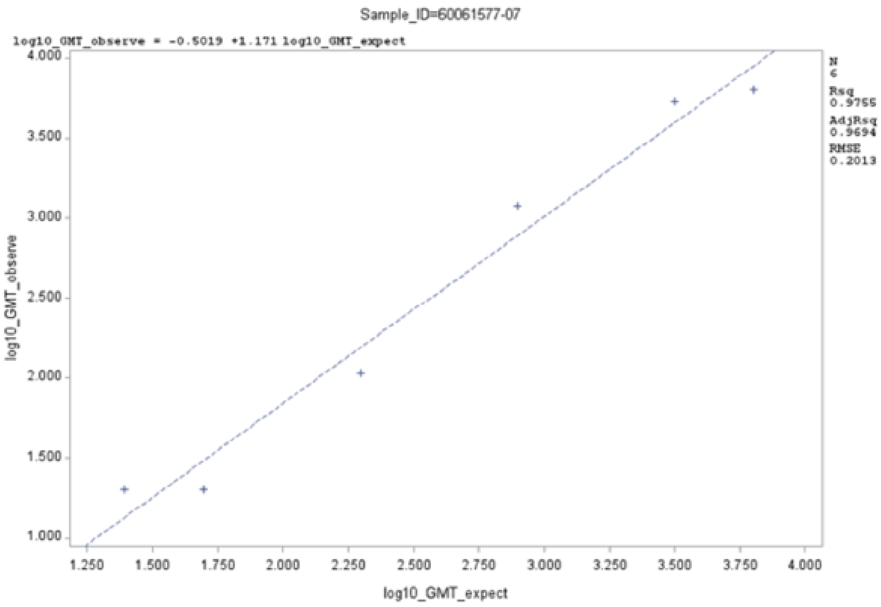

a) Omicron KP.2 subvariant sample ID: 60061577-07

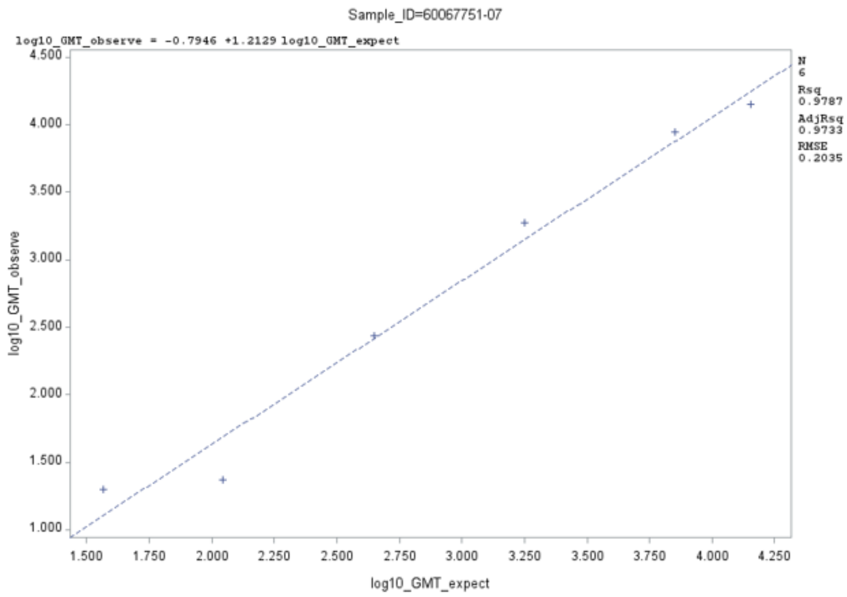

b) Omicron KP.2 subvariant sample ID: 60067751-07

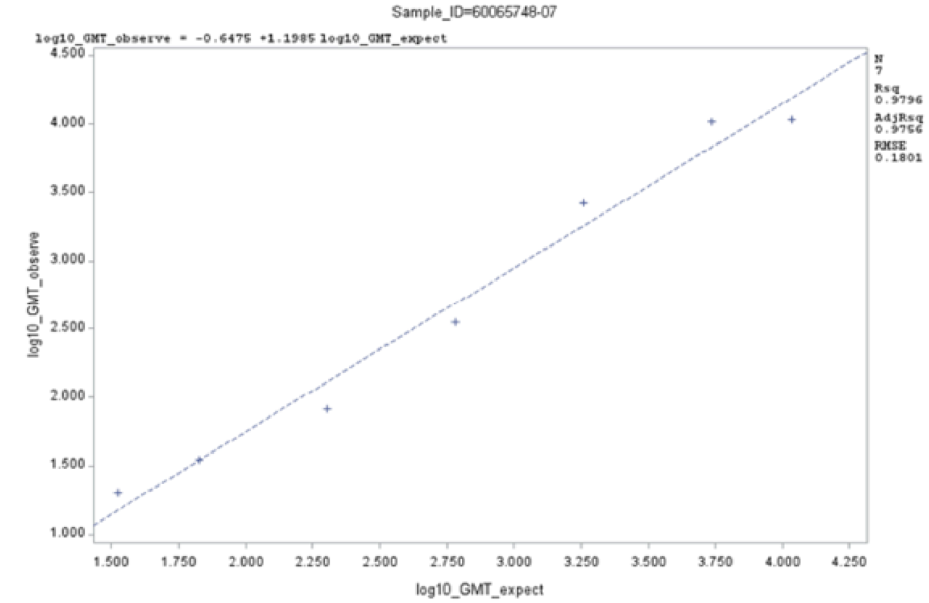

c) Omicron KP.2 subvariant sample ID: 60065748-07

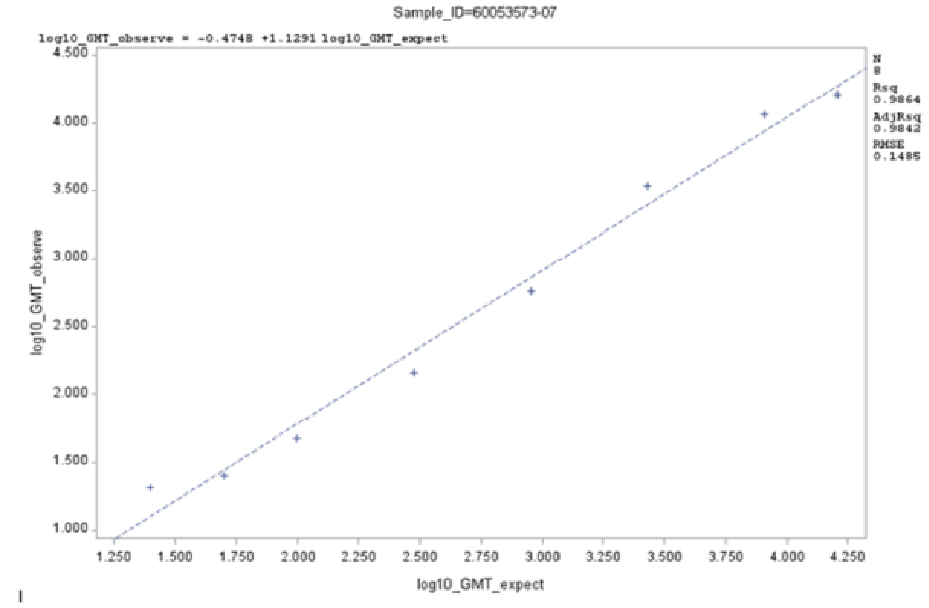

d) Omicron KP.2 subvariant sample ID: 60053573-07

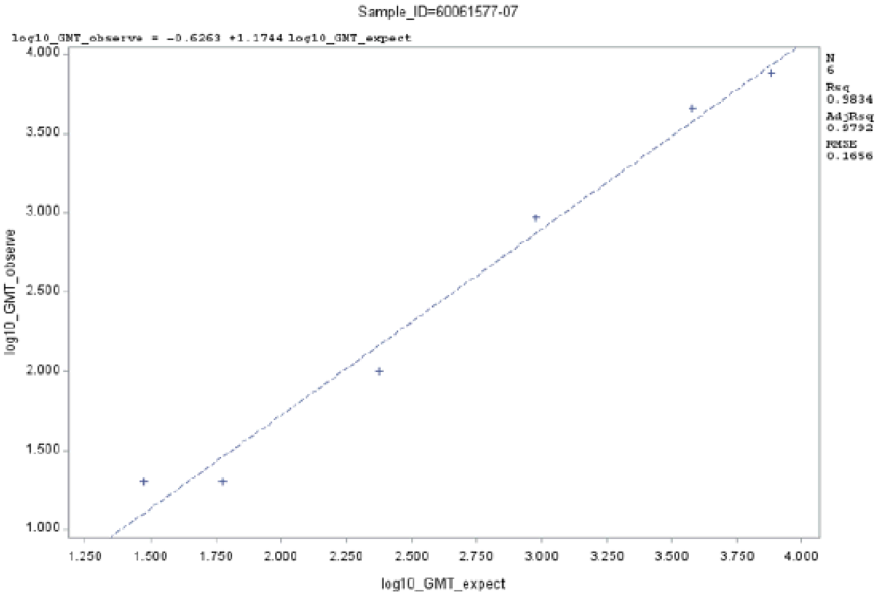

e) Omicron KP.3 subvariant sample ID: 60061577-07

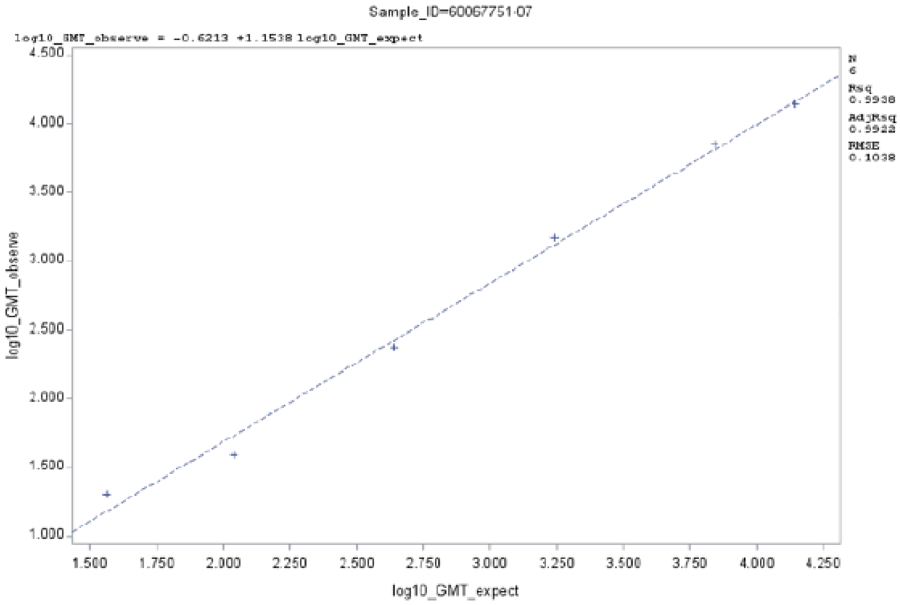

f) Omicron KP.3 subvariant sample ID: 60067751-07

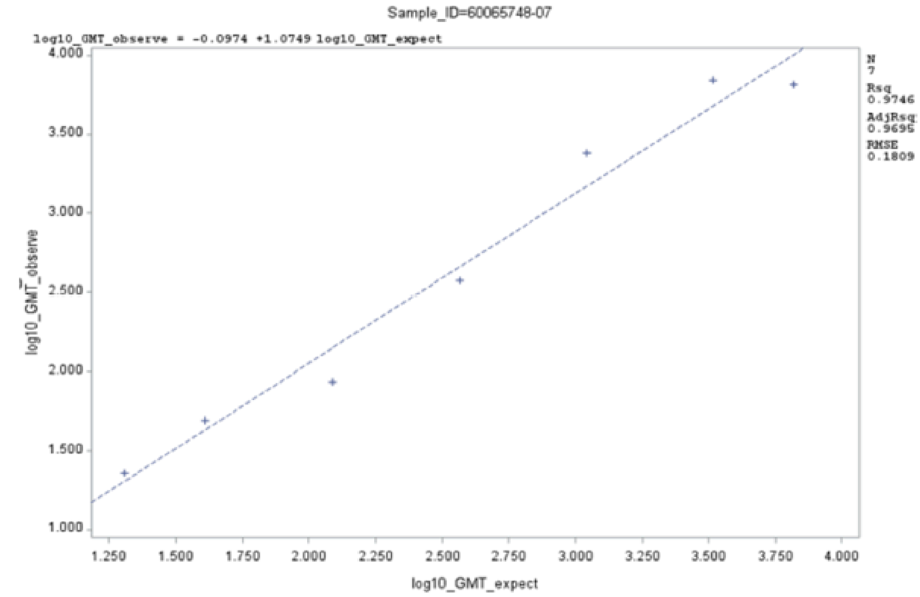

g) Omicron KP.3 subvariant sample ID: 60065748-07

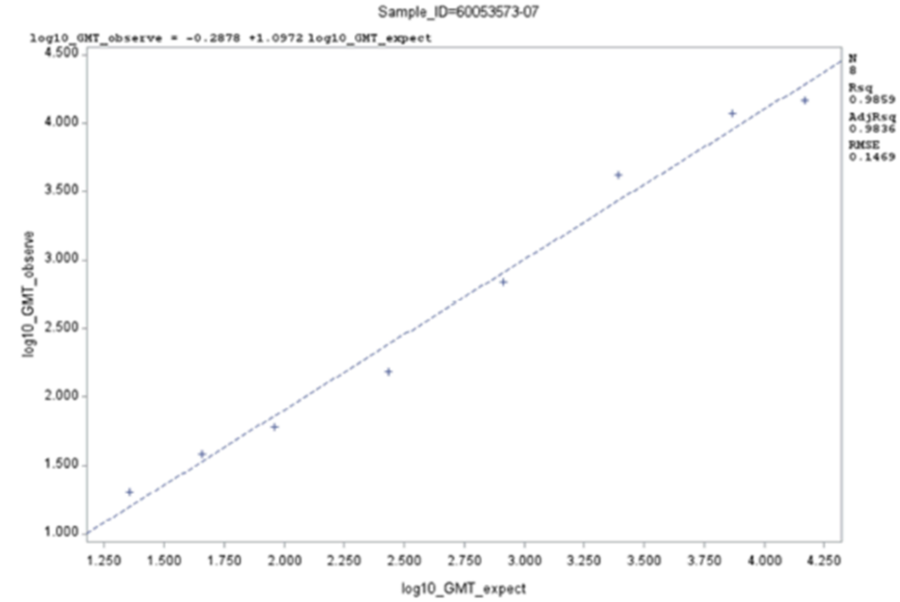

h) Omicron KP.3 subvariant sample ID: 60053573-07

Figure S2. Linear regression plots of PNT GMTs against Omicron KP.2 and KP.3 subvariants – non-matched ('heterologous') JN.1 clinical sera. Abbreviations: GMT, geometric mean titer; ID, identification; PNT, pseudovirus neutralization; R2, coefficient of determination.
